# Supplementary material for: Estimating food resource availability in arid environments with Sentinel 2 satellite imagery
Source: PeerJ. 2020 May 26;8:e9209. doi: 10.7717/peerj.9209 (PMC7258894; doi:10.7717/peerj.9209)
Supplement: Table S1 — Summary of vegetation composition of the two clusters ‘grass’ and ‘shrub’ based on field measures taken in October 2016 (each genus cover, Enneapogon seed productivity and total vegetation cover), as proportion of occurrence for each plant genus, ordered per grass cluster. Pease note that the bare ground cluster was built manually, based on total vegetation cover to be less than 10% (hence is not shown here). The vegetation composition types (Forb, Grass, Shrub and Tree) and the species indicator analysis test (association statistic and p value) are also specified. Significant values from permutation tests represents indicator species for each cluster. [file peerj-08-9209-s001.docx]

**Table S1** Summary of vegetation composition of the two clusters ‘grass’ and ‘shrub’ based on field measures taken in October 2016 (each genus cover, *Enneapogon* seed productivity and total vegetation cover), as proportion of occurrence for each plant genus, ordered per grass cluster. Pease note that the bare ground cluster was built manually, based on total vegetation cover to be less than 10% (hence is not shown here). The vegetation composition types (Forb, Grass, Shrub and Tree) and the species indicator analysis test (association statistic and p value) are also specified. Significant values from permutation tests represents indicator species for each cluster.

| **Plant genus** | **Cluster Grass** | **Cluster Shrub** | **Type** | **Cluster** | **Association statistic** | **p** |
| --- | --- | --- | --- | --- | --- | --- |
| ***Plantago*** | 1 | 0 | Forb | grass | 0.42 | 0.565 |
| ***Poaceae spp.*** | 1 | 0 | Grass | grass | 0.37 | 0.59 |
| ***Sporobolus*** | 1 | 0 | Grass | grass | 0.37 | 0.515 |
| ***Sclerolaena*** | 1 | 0 | Shrub | grass | 0.74 | **0.055** |
| ***Enneapogon*** | 1 | 0 | Grass | grass | 0.72 | **0.045** |
| ***Rhodanthe*** | 0.98 | 0.02 | Forb | grass | 0.92 | **0.005** |
| ***Calotis*** | 0.95 | 0.05 | Forb | grass | 0.33 | 0.885 |
| ***Maireana*** | 0.95 | 0.05 | Shrub | grass | 0.62 | 0.335 |
| ***Chenopodium*** | 0.67 | 0.33 | Shrub | shrubs | 0.31 | 1 |
| ***Erodium*** | 0.67 | 0.33 | Forb | shrubs | 0.31 | 1 |
| ***Pimelea*** | 0.67 | 0.33 | Forb | shrubs | 0.31 | 1 |
| ***Carrichtera*** | 0.67 | 0.33 | Forb | shrubs | 0.31 | 0.73 |
| ***Senna*** | 0.35 | 0.65 | Shrub | shrubs | 0.62 | **0.02** |
| ***Atriplex*** | 0.33 | 0.67 | Shrub | shrubs | 0.50 | 0.065 |
| ***Tetragonia*** | 0.33 | 0.67 | Forb | shrubs | 0.62 | **0.015** |
| ***Medicago*** | 0.17 | 0.83 | Forb | shrubs | 0.90 | **0.005** |
| ***Senecio*** | 0.14 | 0.86 | Forb | shrubs | 0.74 | **0.005** |
| ***Acacia*** | 0.14 | 0.86 | Tree | shrubs | 0.83 | **0.005** |
| ***Rostraria*** | 0.11 | 0.89 | Grass | shrubs | 0.75 | **0.005** |
| ***Myoporum*** | 0 | 1 | Tree | shrubs | 0.76 | **0.005** |
