# Supplementary material for: Estimating food resource availability in arid environments with Sentinel 2 satellite imagery
Source: PeerJ. 2020 May 26;8:e9209. doi: 10.7717/peerj.9209 (PMC7258894; doi:10.7717/peerj.9209)
Supplement: Table S2 — Vegetation indices calculated from Sentinel 2 imageries with relative formulas, native spatial resolution. ‘ ρ NIR’ represents the near- infrared (0.84 µm) and ‘ ρ red’ represents the red (0.66 µm) wavelengths respectively. [file peerj-08-9209-s002.docx]

**Table S2** Vegetation indices calculated from Sentinel 2 imageries with relative formulas, native spatial resolution. ‘ρ NIR’ represents the near- infrared (0.84 μm) and ‘ρ red’ represents the red (0.66 μm) wavelengths respectively.

| **Index** | **Formula** | **Spatial resolution** | **Reference** |
| --- | --- | --- | --- |
| MSAVI_2_ | \| $\frac{2\rho NIR+1-\sqrt{{(2\rho NIR+1)}^{2}-8(\rho NIR-\rho red)}}{2}$ \| \| --- \| | 10 x 10m | Qi 1994 |
| NDVI | $\frac{\rho NIR-\rho red}{\rho NIR+\rho red}$   \|  \| \| --- \| | 10 x 10m | Tucker 1979 |

**References of Table S2:**

Qi, J., Chehbouni, A., Huete, A.R., Kerr, Y.H., Sorooshian, S., 1994. A modified soil adjusted vegetation index. Remote Sens. Environ. 48, 119–126.

Tucker, C.J., 1979. Red and photographic infrared linear combinations for monitoring vegetation. Remote Sens. Environ. 8, 127–150.
