# Supplementary material for: Estimating food resource availability in arid environments with Sentinel 2 satellite imagery
Source: PeerJ. 2020 May 26;8:e9209. doi: 10.7717/peerj.9209 (PMC7258894; doi:10.7717/peerj.9209)
Supplement: Table S3 — Summary of paired Wilcoxon’s signed rank tests to test for differences between cluster subsets in Enneapogon seed-productivity and the total vegetation cover (variables, n=15). Each cluster subset (Pairs), coefficient test (Z) and significance (P) are specified for each comparison. Statistically significant values were Bonferroni adjusted for multiple comparisons and are marked in bold [file peerj-08-9209-s003.docx]

**Table S3** Summary of paired Wilcoxon’s signed rank tests to test for differences between cluster subsets in *Enneapogon* seed-productivity and the total vegetation cover (variables, n=15). Each cluster subset (Pairs), coefficient test (Z) and significance (P) are specified for each comparison. Statistically significant values were Bonferroni adjusted for multiple comparisons and are marked in bold.

| **Variable** | **Pairs (cluster subset)** | **Z** | **P** |
| --- | --- | --- | --- |
| *Enneapogon* seed-productivity | Bare-Grass | 0.41 | 0.11 |
|  | Bare-Shrub | 0.18 | 0.47 |
|  | Shrub-Grass | 0.56 | **0.03** |
| Total vegetation cover | Bare-Grass | 0.55 | **0.03** |
|  | Bare-Shrub | 0.55 | **0.03** |
|  | Shrub-Grass | 0.28 | 0.28 |
