# Supplementary material for: Estimating food resource availability in arid environments with Sentinel 2 satellite imagery
Source: PeerJ. 2020 May 26;8:e9209. doi: 10.7717/peerj.9209 (PMC7258894; doi:10.7717/peerj.9209)
Supplement: Table S4 [file peerj-08-9209-s004.docx]

**Table S4** Summary of the logit estimates from the GLMMs of the relationship between the two vegetation indices (VI) and the response variable (proportion of *Enneapogon* with seeds) for each month.

| **VI** | **Month** | **Estimated proportion of *Enneapogon* with seeds** |
| --- | --- | --- |
| MSAVI_2_ | October | (2.66-0.67)+(-65.32+78.28)*MSAVI_2_ |
|  | December | 2.66+(-65.32)*MSAVI_2_ |
|  | January | (2.66+2.14)+(-65.32-69.15)*MSAVI_2_ |
| NDVI | October | (2.75-0.65)+(-30.76+37.32)*NDVI |
|  | December | 2.75+(-30.76)*NDVI |
|  | January | (2.75+2.96)+(-30.76-47.55)*NDVI |
