# Supplementary figures and images for: Estimating food resource availability in arid environments with Sentinel 2 satellite imagery
Source: PeerJ. 2020 May 26;8:e9209. doi: 10.7717/peerj.9209 (PMC7258894; doi:10.7717/peerj.9209)

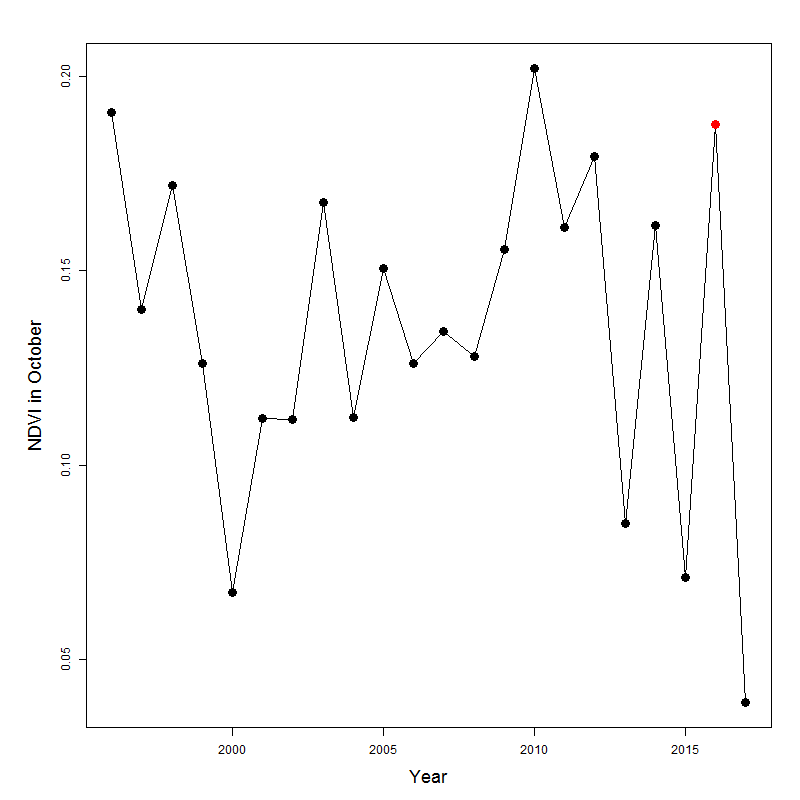

Supplement: Figure S1 — NDVI of October months from 1996 to 2017 (in red October 2016, the period in study) of Gap Hill paddock in Fowlers Gap Research Station, extrapolated by the NDVI maps provided by Australian Bureaux of Meteorology (averaged at 5 km spatial resolution) and processed from Advance Very High Resolution Radiometer (AVHRR, NOAA technology). [file peerj-08-9209-s005.png]
